# Glymphatic System Activity and Brain Morphology in Patients With Psychogenic Non-epileptic Seizures

**DOI:** 10.7759/cureus.53072

**Published:** 2024-01-27

**Authors:** Miho Ota, Daichi Sone, Yoko Shigemoto, Yukio Kimura, Hiroshi Matsuda, Noriko Sato

**Affiliations:** 1 Neuropsychiatry, University of Tsukuba, Tsukuba, JPN; 2 Radiology, National Center of Neurology and Psychiatry, Kodaira, JPN

**Keywords:** voxel-based morphometry, psychogenic non-epileptic seizures, magnetic resonance imaging, glymphatic system, diffusion tensor image analysis along the perivascular space

## Abstract

Background: To clarify the neural correlates underlying psychogenic non-epileptic seizures (PNES), we compared glymphatic system activity between patients with PNES and healthy participants using diffusion tensor imaging (DTI)-analysis along the perivascular space (ALPS) method.

Methods: The DTI scans were acquired from 16 patients with PNES and 25 healthy participants. We computed the DTI-ALPS index as an index of glymphatic system function and estimated the disease-related changes in the DTI-ALPS index and brain structures in PNES patients.

Results: There were no significant differences in the DTI-ALPS index between patients with PNES and healthy participants. On the other hand, patients with PNES had decreased fractional anisotropy values in the bilateral posterior cingula, a higher mean diffusivity value around the left insula, and a lower gray matter volume in the bilateral amygdalae compared with healthy participants.

Conclusions: Patients with PNES exhibited an impairment of white matter integrity and a reduction of gray matter volume, but no glymphatic-system changes. These findings will play a significant role in our comprehension of this complex illness.

## Introduction

Epilepsy is one of the most common brain diseases in the world. However, determining the etiology of epilepsy is difficult because so many factors play a role in the seizures. Psychogenic non-epileptic seizures (PNES) are characterized by episodes that resemble epileptic seizures but are not caused by epileptic discharges in the brain [1]. It is difficult to differentiate PNES from epilepsy and is often misdiagnosed with epilepsy [2]; PNES was considered among the top three neuropsychiatric problems by a consensus clinical practice statement in 2011 [3].

A few neuroimaging studies focusing on PNES have investigated subtle imaging abnormalities. One group reported increased fractional anisotropy (FA) in left hemisphere structures containing the uncinate fasciculus, corona radiata, superior temporal gyrus, and capsules in patients with PNES using whole-brain tract-based spatial statistics (TBSS) [4]. On the other hand, another investigation using TBSS analysis found widespread increases in mean diffusivity (MD) and decreases in FA value in PNES patients [5]. A study employing neurite orientation dispersion and density indices (NODDI) analysis showed that, compared to patients with traumatic brain injury (TBI), PNES patients had lower FA values and neurite density and higher MD for clusters within the uncinate fasciculus, cingulum bundle, corticospinal tract pathways, and fornix/stria terminalis [6]. Cortical thickness analysis in patients with PNES also revealed abnormal cortical volume change in many regions, including the posterior cingulum [7]. Functional MRI (fMRI) studies also revealed amygdala abnormalities [8] and default-mode network regions, including the posterior cingulum [9], in patients with PNES. However, the results derived from structural and functional MRI studies were somewhat controversial.

Previous studies found that the glymphatic system circulates cerebrospinal fluid (CSF) via the brain parenchyma and facilitates the transport of interstitial solutes [10]. The Virchow-Robin space, a part of the glymphatic system, is known to be enlarged by epilepsy [11]. Some researchers have suggested that proinflammatory mediators in patients with epilepsy cause dysfunction of the endothelial tight junctions, which in turn leads to impairment of the blood-brain barrier, abnormal exchange between the CSF and interstitial fluid, and finally changes to the glymphatic system [12-14]. Lately, diffusion tensor imaging (DTI)-analysis along the perivascular space (ALPS) conducted using diffusional imaging data has gained notice because this indicator can approximate the activity of the glymphatic system with a high degree of reproducibility [15-16]. Some studies focused on the relation between the DTI-ALPS index and epilepsy symptoms and showed a decrease in the DTI-ALPS index in patients with such symptoms [12-14], and one study found that glymphatic system function was recovered in patients with temporal lobe epilepsy after successful anterior temporal lobectomy [17]. However, there has been no DTI-ALPS study focusing on PNES until recently. An improved understanding of the neurological basis of PNES and its differences from epilepsy as revealed by DTI-ALPS might be useful for the development of biomarkers.

In this investigation, we first made a comparison of the DTI-ALPS index between healthy participants and patients with PNES, and then we estimated the differences in the white matter integrity and the regional gray matter volumes between them. Our hypothesis was that the DTI-ALPS gauge, a barometer for the activity of the glymphatic system, might be changed in PNES as in other epileptic diseases.

## Materials and methods

Participants

We retrospectively enrolled patients with PNES who took an MRI scan at our hospital from January 2012 to December 2017. The inclusion criteria were: (1) PNES is checked on video-electroencephalogram (EEG) monitoring; and (2) there are no epileptiform seizures or discharges on video-EEG monitoring [18]. Patients were excluded if they had severe head trauma, meningitis, a significant medical history of acute encephalitis, or ischemic encephalopathy, or if they met the criteria for low intelligence quotient (i.e., <69) estimated by the Wechsler Adult Intelligence Scale III. Sixteen patients met the criteria and took part in the study. Twenty-five age- and sex-matched normal participants also took part in this study as the control subjects. Participants were excluded if they had a serious head injury, a history of central nervous system (CNS) illness, or if they met the criteria for substance-related disorders.

Ethics statement

The study adhered to the Declaration of Helsinki and was granted a license from the ethics committee of the National Center of Neurology and Psychiatry, Kodaira, Japan (approval number: A2022-084). Each of the control participants provided written informed consent after the study was explained to them. Our local ethics committee waived the requirement of informed consent from the patients due to the retrospective nature of the study.

Magnetic resonance imaging study

The MRI images were acquired from a 3-Tesla MR scanner (Achieva, Philips Medical Systems, Best, Netherlands) using a 32-channel coil. A three-dimensional (3D) T1-weighted sequence was performed in the sagittal plane (acquisition image resolution, 0.68 × 0.68 × 0.6 mm; repetition time (TR): 7.12 msec; echo time (TE): 3.4 msec; number of signals acquired: 1; flip angle: 10˚). For the diffusional imaging, axial DTI was recorded (acquisition image resolution: 3 × 3 × 3 mm; TR/TE: 5798/61.5 msec; flip angle: 10˚; number of signals acquired: 2; number of diffusion directions: 15; b = 1000 s/mm^2^), and another one was b = 0 s/mm^2^.

Estimation of DTI-ALPS index

The DTI-ALPS index was measured as described previously [15]. First, we took the denoise step and corrected Gibbs ringing artifacts by using MRItrix3 (https://www.mrtrix.org/). Second, susceptibility distortions were corrected using the Synb0-DisCo approach [19]. Third, the b = 0 map of each participant was co-registered to the “EPI.nii” image, the standard template in SPM12 software run on Matlab R2017b (Math Works, Natick, MA), to correct the direction of the brain. Fourth, we compensated image distortion due to field gradient eddy currents using FMRIB Software Library (FSL, Analysis Group, FMRIB, Oxford, UK) 6.0.3 software package (https://fsl.fmrib.ox.ac.uk/fsl/fslwiki/FslInstallation) and matched the position of raw DTI images linearly to the coregistered b = 0 image. Fifth, the b-vector gradient matrix was rotated to fit the changes made in the fourth step by using the fdt_rotate_bvecs tool, which was the command in FSL. Sixth, individual color-coded vector maps, diffusivity maps in the direction of the x-, y-, and z-axis, FA, and MD maps were created. Finally, we put spherical regions of interest (ROIs) with a diameter of 5 mm on the bilateral projection fibers and association fiber tracts. We averaged both DTI-ALPS indices to decrease the multiple comparisons.

Magnetic resonance imaging data processing

We normalized each 3D-T1 image using VBM8 (http://dbm.neuro.uni-jena.de/vbm8) to evaluate the regional gray matter volume. Normalized individual images were smoothed with a full width at half maximum (FWHM) = 12 mm.

For the sake of FA and MD map comparisons between PNES and healthy controls, we normalized the individual FA maps to the “EPI.nii”. Then, the normalized FA maps were averaged to make the standard FA template. The standard FA template was smoothed by using a 6 mm FWHM Gaussian kernel, and the individual FA images were normalized again to the standard FA template. The transformation matrix was applied to the individual MD maps. Finally, the 6 mm FWHM was enrolled here for smoothing individual FA and MD maps.

Statistics

A chi-square test was used to determine differences in sex, and a two-sample t-test was used to assess differences in age between two diagnostic groups. Differences in the DTI-ALPS index between the two groups were analyzed using analysis of covariance (ANCOVA) controlling for sex and age. We set our significance level at p <0.05 after the Bonferroni correction. Data were tested using IBM SPSS Statistics for Windows, ver. 23.0 (IBM Corp., Armonk, NY).

Voxel-based morphometry (VBM) analyses were performed to estimate the volume differences of gray matter between healthy participants and patients with PNES using SPM12. Age and sex were controlled for the analyses. The statistical threshold of significant difference was set at a voxel level of p <0.001 (uncorrected) and made at the cluster level using p <0.05 with family-wise error correction for multiple comparisons.

As for the FA and MD maps, differences in FA and MD values between them were analyzed using SPM12 adjusted for sex and age. We masked the statistical results by using the standard white matter template. The statistical threshold for a significant difference was set at a voxel level of p <0.001 (uncorrected) and a cluster level of p <0.05 (uncorrected).

## Results

Participant demographics and clinical characteristics are listed in Table 1.

**Table 1:** Clinical characteristics of the participants PNES: psychogenic non-epileptic seizures; DTI-ALPS: diffusion tensor image analysis along the perivascular space

	Healthy participants		Patients with PNES		p-value
Male:Female	6:19		3:13		0.69
Age (years)	29.9 ± 6.9		29.5 ± 9.0		0.88
Onset of illness (years)	(-)		22.8 ± 10.0		(-)
Bilateral DTI-ALPS	1.51 ± 0.15		1.46 ± 0.14		0.30

**Figure 1:**
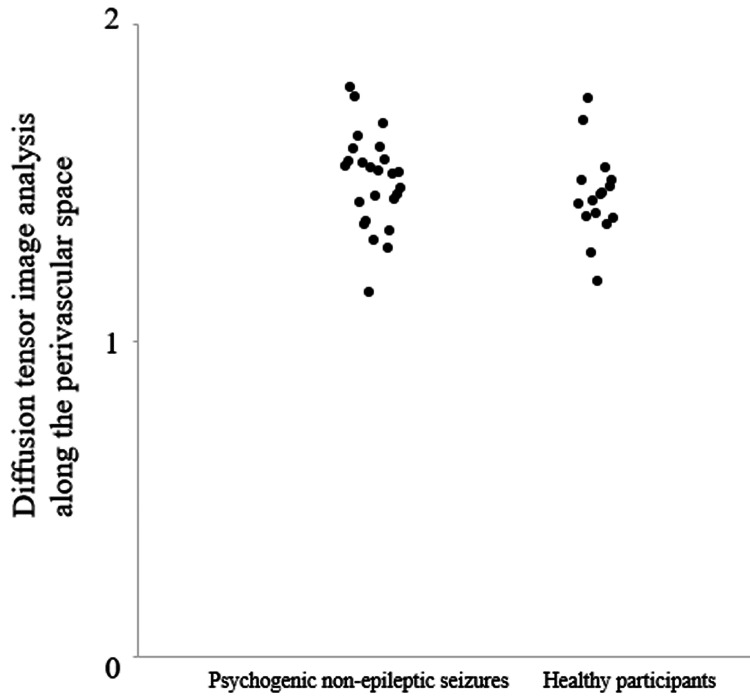
The DTI-APLS index values for patients with PNES and healthy participants The scatter plot shows the DTI-APLS indices of the two diagnostic groups. There was no significant difference between them. DTI-ALPS: diffusion tensor image analysis along the perivascular space; PNES: psychogenic non-epileptic seizures

There were no significant differences in age or sex between the patients with PNES and healthy participants. Regarding the DTI-ALPS index, there was no significant difference between the patients with PNES and healthy participants by ANCOVA (p = 0.310, F = 1.062) controlling for age and gender (Figure 1).

**Figure 2:**
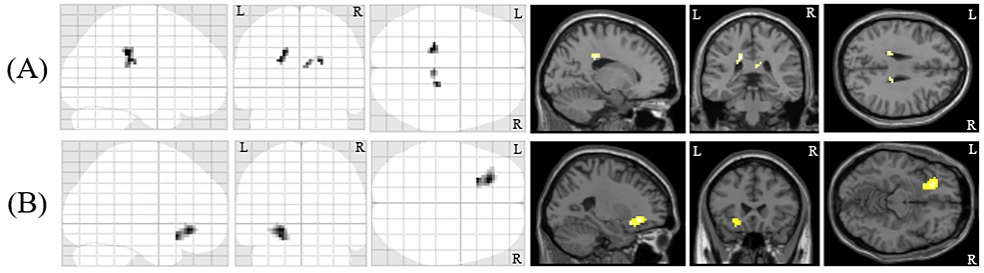
Differences in FA and MD values between patients with PNES and healthy subjects. (A) The patients with PNES showed lower FA values in the bilateral posterior cingula compared with the healthy subjects. (B) The patients with PNES showed higher MD values around the insula compared with the healthy subjects. The background images are “single_subj_T1” images, which are regarded as one of the anatomically standardized images in SPM12. R: right side, and L: left side; FA: fractional anisotropy; MD: mean diffusivity; PNES: psychogenic non-epileptic seizures

Next, we evaluated the differences in FA values between the patients with PNES and healthy participants using SPM12. The FA values of the patients with PNES in the bilateral posterior cingulate regions were decreased significantly (left posterior cingulate region: cluster size = 30 voxels, cluster-level p value = 0.021, voxel T value = 4.13, coordinate of voxel in Talairach space (x, y, z) = (-21, -31, 25); right posterior cingulate region: cluster size = 23 voxels, cluster-level p-value = 0.040, voxel T-value = 3.97, coordinate of voxel (x, y, z) = (18, -28, 25)), compared with those of healthy participants (Figure 2A).

There were no significant increases in FA value in patients with PNES. On the other hand, the MD values of the patients with PNES around the left insula region were significantly increased (cluster size = 95 voxels, cluster-level p-value = 0.003, voxel T-value = 5.55, coordinate of voxel (x, y, z) = (-21, -31, 25), right posterior cingulate region: cluster size = 23 voxels, voxel T-value = 3.97, (x, y, z) = (-24, 29, 8)), compared with those of healthy participants (Figure 2B). We did not find any significant decreases in MD values in patients with PNES.

**Figure 3:**
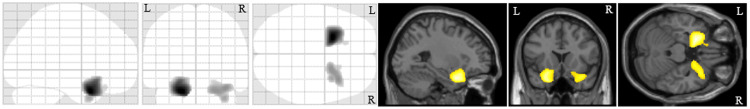
Regional gray matter volume differences between patients with PNES and healthy subjects. The patients with PNES showed significantly smaller gray matter volumes in bilateral amygdalae compared with the healthy subjects. The background images are “single_subj_T1” images, which are regarded as one of the anatomically standardized images in the SPM12. R: right side, and L: left side; PNES: psychogenic non-epileptic seizures

Lastly, we measured the volume differences in gray matter between them. We detected significant regional volume reductions in bilateral amygdalae (left: cluster size = 2921 voxels, cluster-level p-value = 0.006 (familywise error rate (FWE) corrected), voxel T-value = 5.79, coordinate of voxel (x, y, z) = (-20, 9, -29); right: cluster size = 1582 voxels, cluster-level p-value = 0.046 (FWE corrected), voxel T-value = 3.74, coordinate of voxel (x, y, z) = (23, 8, -27)) in patients with PNES compared with those of healthy participants (Figure 3).

There were no significant regional gray matter volume increases in patients with PNES.

## Discussion

In this study, we focused on the glymphatic system in patients with PNES and found that the DTI-ALPS indices of the patients with PNES were not significantly different from those of the healthy participants. This point might suggest that there was no difference in glymphatic system function between them. Further, we detected bilateral amygdalae shrinkage and FA reduction in the posterior cingula and an increase in MD value around the insula of the PNES patients. To our knowledge, this is the first study to estimate the glymphatic system activity in patients with PNES using DTI-ALPS.

We found no difference in the DTI-ALPS index between our patients with PNES and healthy subjects. Some studies have reported glymphatic system dysfunction in epileptic patients [11-14, 17], and others have suggested that the proinflammatory mediators in patients with epilepsy disrupt the blood-brain barrier and thereby damage the glymphatic system [12-14]. Psychogenic non-epileptic seizures are epilepsy-like seizures without epileptic discharges in the brain [1], and unlike in epilepsy, the neurological basis of PNES might not disturb the blood-brain barrier. Without such disturbance, it may be that the glymphatic system in PNES remains intact.

Some studies using structural MRI have detected microstructural changes in the posterior cingulum [4-6], while others using fMRI have found that PNES patients exhibit abnormal activity in the posterior cingulum and other default mode network (DMN) regions [9]. Alterations in the circulation of DMN have been posited to cause changes in consciousness and self-awareness [20], which could lead to modified self-perception during psychogenic events such as PNES [21]. As for the amygdala, one study found that patients with PNES exhibited a decreased response to stress fMRI in the bilateral amygdala [9]. Previous neuroimaging studies focusing on PNES suggested that affect-related areas like the amygdala may disrupt emotional shift and awareness [22], and these points might explain the abnormal emotional processing in PNES patients. In addition, two PNES studies found abnormalities in the uncinate fasciculus [4, 6]. The uncinate fasciculus is an important neural pathway that connects medial prefrontal regions and the limbic system, including the hippocampus and amygdala [23-24], which can serve as an impetus to memory and emotional processes [25]. We found a significant increase in MD value around the insula, where the uncinate fasciculus tract passes through. Our finding might be derived from the microstructural change in the uncinate fasciculus tract.

This study contained some limitations. At first, there were relatively few participants in the study. However, the structural changes of the patients with PNES reached the level of statistical significance, and future research with a larger sample size might confirm the results. Second, it is known that some patients with PNES also experience epilepsy [26]. We therefore cannot conclude that patients with a low DTI-ALPS index do not have PNES. Lastly, the FA change in our patients with PNES was observed only in the posterior cingula. Some DTI studies focusing on PNES investigated diffuse imaging abnormalities calculated by TBSS analysis [4-5]. Lee et al. undertook dMRI by using a different MRI machine with a different b value, and thus their images might not be directly comparable with ours. Further, although the skeleton projection of TBSS was adopted to compensate for local registration errors, this method involves many compromises [27]. For example, TBSS preferentially detects changes in diagonally oriented bundles because skeletonized diagonal neural pathways are thicker in voxel space than vertical or horizontal tracts [28]. In consideration of these points, our original FA template designed to normalize these images might yield more plausible results in terms of PNES-related changes.

## Conclusions

There were no differences in glymphatic system activity between the patients with PNES and healthy participants, as estimated by the DTI-ALPS method. Further, patients with PNES showed FA reduction in the posterior cingulum, volume reductions in the amygdala, and MD increase around the insula, where the uncinate fasciculus tract passes through. It is known that the uncinate fasciculus plays an important role in the emotional network, and these findings suggest that PNES has a different neurological basis from epilepsy.
